# Giant bilateral angiomyolipomas with spontaneous hemorrhage and inferior vena cava thrombosis in a patient with tuberous sclerosis

**DOI:** 10.11604/pamj.2017.27.223.8379

**Published:** 2017-07-25

**Authors:** Meriem Menany, Safae Khnaba, Bouchaib Radouane, Mohamed Jidal, Touriya Amil, Rachida Saouab

**Affiliations:** 1 Department of Radiology, Military Hospital, Ibn Sina CHU, Rabat, Morocco

**Keywords:** bilateral angiomyolipomas, hemorrhage, fat thrombosis

## Abstract

Renal angiomyolipomas are rare type of benign renal neoplasm. They are composed of vascular, smooth and fat elements and can be associated to phacomatosis as Tuberous Sclerosis disease. Symptomatic presentation is most frequently spontaneous retroperitoneal hemorrhage, which can be fatal. The risk of bleeding is proportional to the size of the lesion (>4 cm of diameter). Typical angiomyolipomas are benign but may have alarming properties: nuclear pleomorphism and mitotic activity, extension into the vena cava, and spread to regional lymph nodes without malignant progression. We report a Computed Tomography finding of a rare giant bilateral angiomyolipomas with spontaneous hemorrhage and inferior vena cava thrombus in a patient with tuberous sclerosis, emphasizing the importance of imagery in the positive and etiologic diagnosis.

## Introduction

Angiomyolipoma (AML) is a mesenchymal renal tumor composed of variable proportions of adipose tissue as well as vascular and smooth muscles elements. In patients with tuberous sclerosis, it can be associated with significant morbidity, mostly related to complications from bleeding.

## Patient and observation

A 28 years old woman complained about a persistant left flank pain accompagned by schok. At her first year old, after an epilepsy, radiologic investigations had shown that the patient was suffering of tuberous sclerosis disease. The clinical examination revealed low blood pressure, tachycardia and a voluminous lumbal left mass. The laboratory analysis showed hemoglobin: 6g/dl and creatinine 22mg/dl.

**Figure 1:**
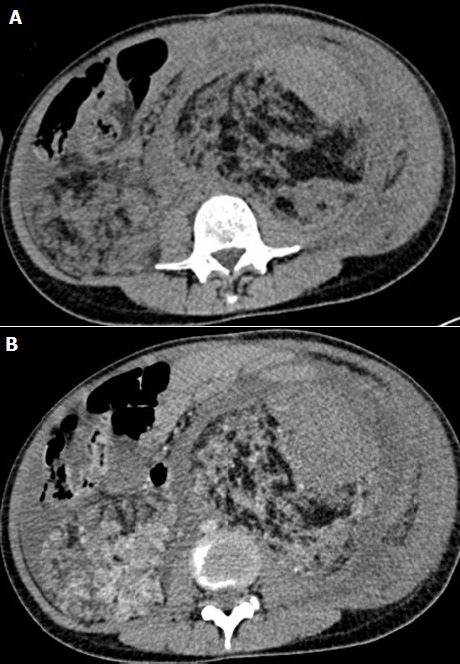
(A,B) axial abdominal computed tomographic scan showing bilateral abdominal masses of fat attenuation that replace and displace renal parenchyma bilaterally, with a lesion of soft tissue attenuation and whorled configuration of hyper attenuating fluid that suggests recent intra-tumoral hemorrhage in the left kidney

**Figure 2:**
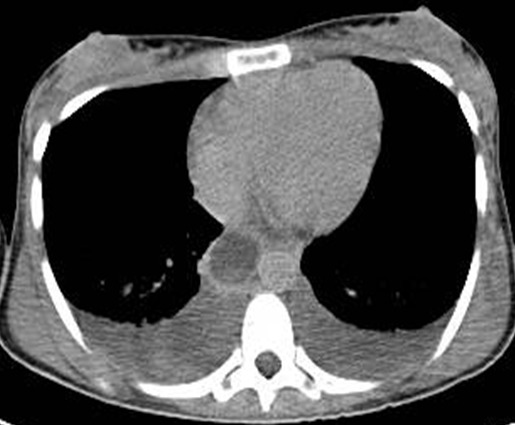
Axial CT scan showing hypo attenuated tumor thrombus in the inferior vena cava and bilateral pleural effusion

**Figure 3:**
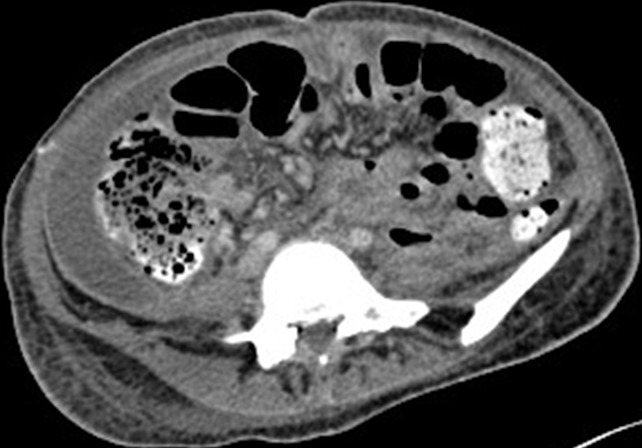
Axial CT scan after embolization and bilateral nephrectomy.

**Figure 4:**
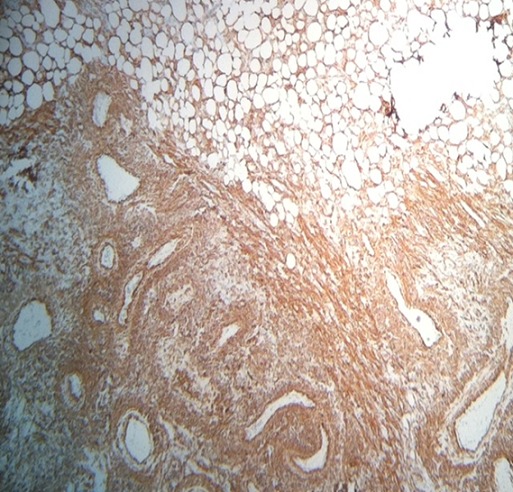
Gross specimen of the left kidney showing angiomyolipomatous proliferation of benign appearance (Ex10)

**Figure 5:**
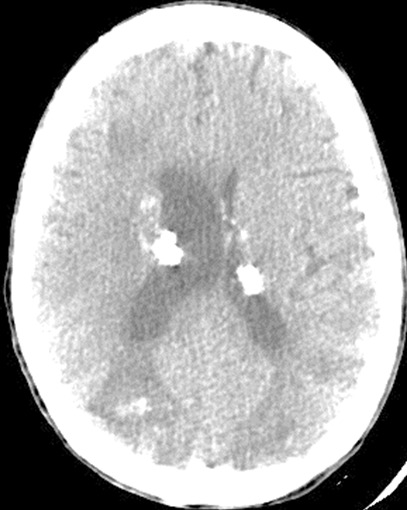
Axial unenhanced CT scan of the brain shows numerous calcified sub ependymal nodules

Abdominal computed tomography (CT) without intra-venous contrast showed large bilateral abdominal masses. Those masses were composed of fat density, thatdistorted renal parenchyma and extended into the pelvis. The left kidney mass was voluminous, measured 46x25x36mm and compressing surrounding organs. It contains a large lesion of soft tissue attenuation, and whorled configuration of hyper attenuating fluid, that suggests recent intra-tumoral hemorrhage. Fatty tumor extension into the inferior vena cava was also noted (Figure 1, Figure 2). Based on the imaging finding, a selective embolization was performed, and a bilateral nephrectomy had been done 8 days later (Figure 3). The histologic evaluation confirmed the presence of adipose tissue, smooth muscle cells and dystrophic vessels (Figure 4). One week later, the patient presents confusion and a brain CT was performed and showed numerous calcified sub ependymal nodules (Figure 5).

## Discussion

Renal angiomyolipoma is uncommon benign tumor; for the general population, this incidence stand between 0.07% and 0.3% with most commonly manifesting in middle aged woman. 20% of angiomyolipomas are associeted with tuberous sclerosis; which is an autosomal dominant phacomatosis, caused by abnormalities on chromosome 9 or 16. Tuberous sclerosis is classically described as the triad of adenoma sebaceum, seizures and mental retardation [1].

Historically, the first histologic description of an angiomyolipoma appeared in litteratture in a 1911. The components of this tumor include mature adipose tissue, dystrophic vessels and smooth muscles cells. In patient with tuberous sclerosis, angiomyolipoma are typically silent. Mostly, 77% of tumors will be smaller than 4 cm and are asymptomatic; 82% of AMLs bigger than 4 cm produce symptoms. The presenting symptoms primarily include fever, nausea, vomiting, pain, palpable mass, hematuria, hypertension, anemia, renal failure, and shock. Retroperitoneal hemorrhage (Wunderlich syndrome) occurs in 50% of patients with tumors bigger than 4 cm, for cases of tubrous sclerosis and numerous confluent AMLs. Urinary tract infection is an uncommon presentation. The extension of a renal AML into the renal vein, IVC, or atrium is a significant point of morbidity with this disease. Only a few cases reports of AML with tumor extension have been published [2].

Many imaging modalities show typical but nonspecific finding. At sonography, renal angiomyolipomas are intensely echogenic and may cause acoustic shadowing [**3**]. They are round or oval cortical tumors; they tend to be well circumscribed, with an echogenicity similar to that of the echogenic renal sinus. Because of their intense echogenicity, angiomyolipomas as small as a few millimeters in diameter may be identified. Less echogenic areas within the tumor are related to hemorrhage, necrosis, or dilated calyces. A reduction of echogenicity in angiomyolipomas is related to a decrease in the quantity of fat and to an increase in the prominence of myogenic components. Doppler ultrasonography may be used to confirm the rare complication of extension into the renal vein and the IVC [2].

The characterization of angiomyolipomas with CT is dependent on spatial resolution and accurate determination of attenuation values; newer spiral scanners meet these criteria. As a result, CT scanning is highly accurate in the characterization and diagnosis of angiomyolipoma lesions [3].

At CT, angiomoyolipomas in TS appears as a well-marginated, cortical, predominantly fat-attenuation mass with heterogeneous soft-tissue attenuation interspersed throughout the lesion. The soft-tissue attenuation may be the result of hemorrhage or fibrosis or a manifestation of the vascular or smooth muscle components of the lesion. When negative attenuation values of less than 20 HU is recorded in renal tumors, angiomyolipomas may be reliably diagnosed in the appropriate clinical setting, and the diagnosis of a renal cell carcinoma can generally be ruled out. However, isolated reports of renal cell carcinoma with demonstrable fat content have appeared in the literature. These renal carcinomas may entrap surrounding perinephric fat or undergo fatty change because of metaplasia. Intratumoral fat is reported in Wilms tumors, oncocytoma, xanthogranulomatous pyelonephritis, renal and retroperitoneal liposarcoma, and teratoma. A false-negative diagnosis is made in the 5% of patients with angiomyolipomas that contain only microscopically visible fat. Angiomyolipomas may calcify and cause the HU value to increase out of the range for fat. However, this effect is rare; significant calcification should prompt the reconsideration of angiomyolipoma as a diagnosis [3–6].

Magnetic resonance imaging does not appear to provide any advantages compared with CT, except when intravenous contrast administration is contraindicated [7]. The characteristic appearances of angiomyolipomas with MRI include variable areas of high signal intensity within the tumor on both T1-weighted and T2-weighted images. On a no enhanced T1-weighted image, high signal intensity is present because of the fat content. On T2-weighted images, the signal remains isointense relative to that of perinephric fat. However, areas of high signal intensity on T1-weighted images are not pathognomonic of fat, and blood and pockets of fluid of high protein content may have a similar appearance. Intratumoral fat is best demonstrated with fat-suppression techniques. The in-phase and out-of-phase T1-weighted imaging technique is extremely sensitive to small quantities of fat. MRI studies may show the rare complication of regional lymph node involvement and invasion of the renal vein and IVC [2].

Traditionally, angiomyolipoma has been diagnosed by comparing T1-weighted images incorporating frequency-selective fat suppression with T1-weighted images without frequency-selective fat suppression. Angiomyolipomas may also be diagnosed using opposed-phase chemical shift artifact [8].

Some urologists request preoperative embolization of the tumor-harboring kidney to decrease/avoid extensive blood loss during surgery and/or to facilitate surgery with huge renal tumors when the renal vessels are difficult to reach. The complications of nephron-sparing surgery related to bleeding or arteriovenous fistulas may be attenuated by arterial embolization.

Oesterling et al. recommended that symptomatic tumors less than 4cm should be observed regularly with CT or ultrasound, whereas those greater than 4cm should be studied by angiography and considered for arterial embolization or surgery. The current management options include observation, embolization, and partial and total nephrectomy. In general, symptomatic masses or masses greater than 8 cm would require special intervention. Beyond the total nephrectomy for patients with highly suspected malignancy, conservative treatment with observation strategy was suggested for small asymptomatic tumors; selective arterial embolization could be the first choice for hemorrhage or rupture and partial nephrectomy for renal sparing strategy [9].

## Conclusion

Renal Angiomyolipoma is a rare benign tumor; it may cause serious complications though spontaneous rupture and fat thrombosis. Our patient had one of the largest bilateral angiomyolipoma ever reported in literature, with a particularity of double complications; spontaneous hemorrhage and extension in inferior vena cava.

## Competing interests

The authors declare no competing interests.
